# Case Report: Efficacy of Reduced Doses of Asfotase Alfa Replacement Therapy in an Infant With Hypophosphatasia Who Lacked Severe Clinical Symptoms

**DOI:** 10.3389/fendo.2020.590455

**Published:** 2020-12-18

**Authors:** Yasuko Fujisawa, Taichi Kitaoka, Hiroyuki Ono, Shinichi Nakashima, Keiichi Ozono, Tsutomu Ogata

**Affiliations:** ^1^ Department of Pediatrics, Hamamatsu University School of Medicine, Hamamatsu, Japan; ^2^ Department of Pediatrics, Faculty of Medicine, Graduate School of Medicine, Osaka University, Osaka, Japan

**Keywords:** hypophosphatasia, mild symptoms, asfotase alfa, hyperphosphatemia, dosage reduction

## Abstract

**Background:**

Hypophosphatasia is a rare bone disease characterized by impaired bone mineralization and low alkaline phosphatase activity. Here, we describe the course of bone-targeted enzyme replacement therapy with asfotase alpha for a female infant patient with hypophosphatasia who lacked apparent severe clinical symptoms.

**Case presentation:**

The patient exhibited low serum alkaline phosphatase (60 U/L; age-matched reference range, 520–1,580) in a routine laboratory test at birth. Further examinations revealed skeletal demineralization and rachitic changes, as well as elevated levels of serum calcium (2.80 mmol/L; reference range, 2.25–2.75 mmol/L) and ionic phosphate (3.17 mmol/L; reference range, 1.62–2.48 mmol/L), which are typical features in patients with hypophosphatasia. Sequencing analysis of the tissue-nonspecific alkaline phosphatase (*TNSALP*) gene identified two pathogenic mutations: c.406C>T, p.Arg136Cys and c.979T>C, p.Phe327Leu. Thus, the patient was diagnosed with hypophosphatasia. At the age of 37 days, she began enzyme replacement therapy using asfotase alpha at the standard dose of 6 mg/kg/week. Initial therapy from the age of 37 days to the age of 58 days substantially improved rickets signs in the patient; it also provided immediate normalization of serum calcium and ionic phosphate levels. However, serum ionic phosphate returned to a high level (2.72 mmol/L), which was presumed to be a side effect of asfotase alpha. Thus, the patient’s asfotase alfa treatment was reduced to 2 mg/kg/week, which allowed her to maintain normal or near normal skeletal features thereafter, along with lowered serum ionic phosphate levels. Because the patient exhibited slight distal metaphyseal demineralization in the knee at the age of 2 years and 6 months, her asfotase alfa treatment was increased to 2.4 mg/kg/week. No signs of deterioration in bone mineralization were observed thereafter. At the age of 3 years, the patient’s motor and psychological development both appeared normal, compared with children of similar age.

**Conclusion:**

This is the first report in which reduced doses of asfotase alfa were administered to an infant patient with hypophosphatasia who lacked apparent severe clinical symptoms. The results demonstrate the potential feasibility of a tailored therapeutic option based on clinical severity in patients with hypophosphatasia.

## Introduction

Hypophosphatasia (HPP) is caused by a loss-of-function mutation in the gene encoding tissue non-specific alkaline phosphatase (TNSALP) (1). Insufficient TNSALP activity leads to extracellular accumulation of its substrates: inorganic pyrophosphate (PPi) and pyridoxal 5′-phosphate (PLP) (1). Elevated extracellular PPi levels inhibit bone mineralization and cause impaired skeletal mineralization (1–3). Reduced phosphorylation of PLP to pyridoxal is associated with vitamin B6-responsive seizures in infants with HPP (1, 4, 5). The most severe forms of HPP (perinatal and infantile) have approximately 50–100% mortality rates during infancy because of multiple fractures and severe respiratory problems (1, 6–8). In contrast, benign prenatal, childhood, adult, and odonto forms involve mild manifestations with relatively favorable clinical progression (1).

Asfotase alfa (AA) is a well-tolerated, safe, and bone-targeted enzyme replacement therapy recently developed for treatment of patients with HPP (6, 8–10). Clinical studies have mainly been performed involving patients with severe forms of HPP; studies involving patients with mild forms of HPP have been limited, such that it remains controversial whether patients lacking life-threatening symptoms should receive AA treatment (11).

Here, we describe a female infant with HPP who lacked apparent clinical symptoms except characteristic demineralization and rachitic changes revealed by X-ray examination. She was treated with AA at the currently approved standard dose, followed by dose reduction. Her skeletal features substantially improved after AA therapy at the standard dose from the age of 37 days to the age of 58 days; treatment with lower doses allowed her to maintain the improved bone mineralization and concurrently achieve satisfactory physical development.

## Case Description

**Figure 1:**
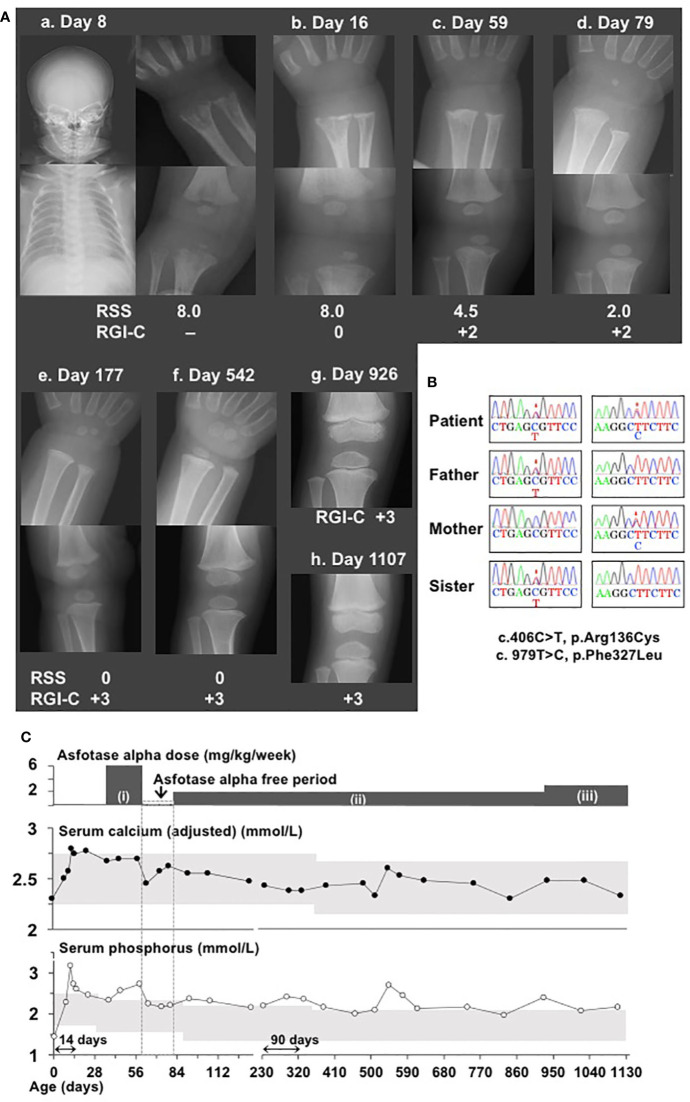
Clinical and molecular findings in a patient with hypophosphatasia. **(A)** X-ray examinations of the wrist and knee showed typical features of hypophosphatasia, whereas the head and chest showed no abnormal features (a and b). X-ray examinations of the wrist and knee after initiation of asfotase alfa (AA) therapy with the standard dose (2 mg/kg, three times per week = 6 mg/kg/week) showed substantial improvement of rickets (c. 59 days of age). Temporary interruption of AA therapy did not worsen skeletal features, as indicated by reduction of Rickets Severity Scale score from 4.5 to 2.0 and maintenance of Radiographic Global Impression of Change rating +2 (d). Lower doses of AA (2 mg/kg/week) maintained normalized skeletal features (e and f). X-ray examination of knee revealed slight distal metaphyseal demineralization (g); increased dose from 2 mg/kg/week to 2.4 mg/kg/week led to recovery of mineralization (h). “Day” represents days of age. **(B)** Electrochromatography showed a compound heterozygous missense mutation of *TNSALP* (c.406C>T, p.Arg136Cys and c.979T>C, p.Phe327Leu) in this patient, as well as a heterozygous missense mutation at c.406C>T in her father and sister, and a heterozygous missense mutation c.979T>C in her mother. Mutations are indicated by asterisks. **(C)** Clinical course of disease in this patient. Upper graph shows changes in serum calcium levels after Payne’s correction (13). Lower graph shows changes in serum ionic phosphate levels. Gray areas indicate age-matched reference ranges. AA therapy at the dose of (i): 6 mg/kg/week, (ii): 2 mg/kg/week, and (iii): 2.4 mg/kg/week. Monitoring frequencies differed among clinical phases, such that monitoring was performed at 4–7-day intervals during pretreatment, initiation of therapy, and interruption of therapy; at 2–4-week intervals during resumption of therapy at a reduced dose; and at 2–3-month intervals during follow-up. Thus, the horizontal line axis from birth to 134 days of age was set to a period of 14 days; from 214 to 1,108 days, it was set to be a period of 90 days. “Day” represents age in days.

A Chinese female infant was born in Japan to non-consanguineous parents at 40 weeks of gestation after an uncomplicated pregnancy. Antenatal sonographic evaluation did not reveal any abnormal born features such as shorten and deformed limbs and hypoechogenic skull seen in skeletal dysplasia. Her birth length, weight, and head circumference were 51.5 cm [+1.0 standard deviation (SD), according to Japanese standards, which were applied because the patient’s family resided in Japan], 3.53 kg (+1.0 SD, according to Japanese standards), and 34 cm (+0.4 SD, according to Japanese standards), respectively. She was hospitalized for mild respiratory distress due to transient tachypnea of the newborn at birth. Routine laboratory tests revealed extremely low alkaline phosphatase (ALP) levels (60 U/L; age-matched reference range, 520–1,580 U/L). Subsequent X-ray examinations showed skeletal demineralization and rachitic changes such as metaphyseal flaring, metaphyseal demineralization, and striking protrusions of radiolucency extending from the metaphyses, all of which are characteristic features of HPP (12); no signs of head or chest-wall deformities were observed (**Figures 1A**, a and b). Further laboratory tests revealed high urine phosphoethanolamine levels (1,902.5 mmol/mg·Cre; age-matched reference range, 7–70 mmol/mg·Cre). Based on the findings, the patient was diagnosed with HPP, presumably the benign prenatal or infantile type. However, some typical features were not observed; these included chest deformities and failure to thrive (signs of infantile HPP) (1, 6, 14), as well as long-bone deformity (a sign of benign prenatal HPP) (1). Very early timing of diagnosis for HPP might be prior to an appearance of failure to thrive symptoms. The patient’s respiratory problems were resolved promptly by transient oxygen administration, suggesting that this symptom was independent of HPP.

## Diagnostic Assessment, Therapeutic Intervention, Follow-Up, and Outcomes

Using leukocyte genomic DNA samples from the patient, sequencing analysis of the coding exons and flanking splice sites of *TNSALP* revealed a compound heterozygous missense mutation comprising c.406C>T, p.Arg136Cys and c.979T>C, p.Phe327Leu; analysis of samples from the patient’s family revealed the heterozygous mutation c.406C>T in her father and elder sister, as well as the heterozygous mutation c.979T>C in her mother (**Figure 1B**). These mutations have been previously identified as pathogenic mutations (15). These results confirmed the diagnosis of HPP in our patient. ALP levels in her father, mother, and sister were 125 U/L (age-matched reference range, 104–338 U/L), 206 U/L (age-matched reference range, 104–338 U/L), and 374 U/L (age-matched reference range, 420–1,130 U/L), respectively. Her parents and sister did not show any manifestations of a disease phenotype.

The patient’s clinical course is summarized in **Figure 1C**. Her serum calcium and ionic phosphate levels during the pretreatment period were elevated to 2.80 mmol/L (age-matched reference range, 2.25–2.75 mmol/L) and 3.17 mmol/L (age-matched reference range, 1.62–2.48 mmol/L), respectively. At the age of 37 days, she began enzyme replacement therapy with AA at a dose of 2 mg/kg three times per week (6 mg/kg/week), which has been currently considered the standard dose. AA was kindly provided by Alexion Pharmaceuticals Inc. (New Haven, CT, USA) for compassionate use, because it had not been approved in Japan. AA therapy immediately normalized serum calcium and ionic phosphate levels. Serum calcium levels generally remained within the reference range throughout the course of treatment. When the patient reached the age of 59 days, X-ray examinations showed remarkable improvement of skeletal features (**Figures 1A**, c), which comprised a reduction of Rickets Severity Scale score (16) from 8.0 to 4.5, as well as enhancement of +2 in the Radiographic Global Impression of Change rating, which is considered to indicate substantial recovery from HPP-associated rickets (17). Notably, serum ionic phosphate returned to a high level (2.72 mmol/L), which exceeded the upper limit for the age-matched reference range. Thus, AA replacement was temporarily discontinued for safety concerns because of this unexpected adverse effect. Interruption of AA therapy until the age of 79 days (an interval of 20 days) resulted in a reduction of serum ionic phosphate levels by 2.18 mmol/L without the reappearance of typical skeletal manifestations (**Figures 1A**, d), along with further reduction in Rickets Severity Scale score from 4.5 to 2.0. At the age of 80 days, the patient’s AA therapy was resumed with a reduced dose (2 mg/kg, once per week). Her serum ionic phosphate levels remained lower than they had been initially, except for a brief elevation observed at the age of 543 days (2.72 mmol/L); notably, these values were slightly above the upper limit of the age-matched reference range (serum ionic phosphate: 1.98–2.46 mmol/L, reference range:1.35–2.27 mmol/L). Lower doses of AA therapy enabled maintenance of improved skeletal features according to the Rickets Severity Scale score (0 point) and Radiographic Global Impression of Change rating (+3, defined as complete or near complete healing rating) (**Figures 1A**, e and f). When the patient reached the age of 926 days, her AA dose was increased from 2 mg/kg/week to 2.4 mg/kg/week, because slight distal metaphyseal demineralization in the knee was detected by X-ray examination (**Figures 1A**, g). No signs of deterioration in bone mineralization were observed thereafter, which was consistent with the +3 Radiographic Global Impression of Change rating at the age of 1,107 days (**Figures 1A**, h). No ectopic calcification or craniosynostosis has been observed so far. Of note, levels of TNSALP substrates (plasma PLP and PPi), which are specific markers of HPP, were examined at Alexion Pharmaceuticals Inc. during the limited period of compassionate use (**Table 1**). At baseline, the patient’s plasma PLP level was elevated, whereas her plasma PPi level was within the reference range. AA therapy led to reduction of PLP levels, although these remained slightly above the upper limit of the reference range, while PPi levels remained within the reference range. At the age of 3 years, the patient demonstrated normal growth with a length of 99.2 cm (+0.4 SD according to Japanese standards) and weight of 15.82 kg (+0.7 SD according to Japanese standards). The patient did not have any dental manifestations throughout the study. The patient’s motor and psychological development were both normal, compared with children of similar age.

**Table 1:** Plasma PLP and PPi levels at baseline and during follow-up.

Dose of AA (mg/kg/week) (period, age (days))	Pretreatment	6 (37-59)	0 (60-79)	2 (80-925)		2.4 (926-1130)
Age (days)	37 (Baseline)		79	134	233	
PLP (RR; 11.76-68.37 ng/mL)	333	NE	254	86	131	NE
PPi (RR; 1.31-5.71 µM)	4.45	NE	3.99	4.11	4.58	NE

AA, asfotase alpha; PLP, pyridoxal 5’-phosphateinorganic pyrophosphate; PPi, inorganic pyrophosphate; RR, reference range; NE, not examined.

This data was not used for adjusting the dose of AA as it was obtained retrospectively.

### Patient Consent

Written informed consent was obtained from the parents of our patient for genetic examination and compassionate use of asfotase alpha. This study was approved by the Independent Ethics Committee of Hamamatsu University School of Medicine.

## Discussion

Although AA therapy has unquestionable benefits for patients with life-threatening HPP, it has remained controversial for use in treatment of patients with less severe symptoms. Recently, the beneficial effects of AA therapy on adult and adolescent patients with mild symptoms have been reported (18). Furthermore, AA therapy for a male infant patient with limited disease manifestation (e.g., poor linear growth, mild limb bowing, and radiographic rickets) resulted in considerable improvement of his symptoms (19). Thus far, conservative management with periodic follow-up is recommended for patients with childhood HPP who exhibit mild or no symptoms; at the onset of any functional limitations, the initiation of AA therapy is recommended (11). Notably, our patient lacked apparent clinical features of HPP; however, substantial X-ray abnormalities were identified in the early neonatal period. The considerable improvement of radiographic features by initial AA therapy suggests that early intervention may have prevented development of further symptoms. However, the longer-term effects of AA remain unknown. Further patient data are needed to determine whether patients without life-threatening symptoms are also appropriate candidates for this therapy.

Lower doses of AA (2 mg/kg/week at 80–926 days of age; 2.4 mg/kg/week at 927–1,107 days of age) maintained favorable improvement of skeletal features, along with satisfactory developmental achievement expected for patients at the age of 3 years (1,107 days of age) (20). The standard dose of AA has been set at 6 mg/kg/week (either 1 mg/kg, six times per week, or 2 mg/kg, three times per week) based on clinical trials mainly in patients with perinatal severe and infantile HPP (8, 12); this dose can be increased to 9 mg/kg/week, depending on the clinical severity in an individual patient. For pediatric patients, adjustments concerning dose reduction have not been reported so far; thus, clinical practice guidelines (21) indicate that there is minimal evidence to support the suitability of dosage reduction during AA therapy. Dose adjustments based on clinical severity could be favorable in the context of minimizing possible side effects. Further careful follow-up is needed to determine our patient’s clinical progression with age.

Our patients carried compound heterozygous mutations in *TNSALP*. One of the identified mutations in our patient, p.Phe327Leu (c.979T>C), has been primarily detected in patients with peripheral benign HPP (22); it has occasionally been detected in patients with odonto, childhood (23), and infantile and childhood (23) HPP, but not in patients with perinatal severe HPP. The p.Phe327Leu mutant TNSALP reportedly retained 72% of wild-type activity (24). Furthermore, patients with the mutation combination of p.Phe327Leu and c.1559delT, which is expected to exhibit extremely low activity (24) show relatively mild clinical manifestations (24). Thus, reports in the literature suggest that this mutation is responsible for mild phenotypes (22, 23, 25), as observed in our patient. The other identified mutation in our patient, p.Arg136Cys (c.406C>T), has been identified in patients with adult (26), odonto (15), or perinatal severe (27) HPP; it is expected to be pathogenic, based on protein function prediction software (27). The loss of activity due to this mutation might be limited due to unchanged hydrogen bonds in the vicinity (27), which may have influenced the clinical presentation in our patient. Together, the relatively mild clinical features in our patient are consistently elucidated by the identified mutations.

Serum calcium and ionic phosphate levels were of particular interest in our patient. Elevated serum calcium and ionic phosphate levels are common in patients with untreated HPP (14, 20). The cause of hypercalcemia in patients with HPP is impaired bone mineralization (1). Thus, AA therapy is the most effective approach to reduce serum calcium levels. Indeed, AA therapy immediately reduced serum calcium levels in our patient; subsequently, normal levels generally persisted. In contrast, the cause of hyperphosphatemia in patients with HPP is partly related to enhanced ratio of renal tubular maximum reabsorption of phosphate to glomerular filtration rate, presumably accompanied by insufficient levels of a phosphaturic peptide, phosphatonin (28). The involvement of phosphatonin insufficiency in patients with HPP is supported by the results of a recent study indicating a low level of fibroblast growth factor 7 (a potent phosphatonin) in pediatric patients with HPP (29). Furthermore, the initial changes of serum phosphate levels in response to AA therapy can either increase or decrease (20); these values typically normalize with treatment, but continued hyperphosphatemia related to AA therapy is regarded as an adverse effect (9), which may lead to worsening of craniosynostosis (30). In our patient, serum phosphate levels remained above the upper limit of the reference range throughout the study, regardless of the use of AA therapy. Considering that temporary interruption of therapy appeared to reduce serum ionic phosphate levels, continued hyperphosphatemia after the initiation of AA therapy in our patient was presumably related to the use of AA. However, the underlying mechanism of hyperphosphatemia in patients receiving AA therapy remains unknown; the long-term risks associated with AA therapy are unclear thus far. Although, no apparent adverse effects such as ectopic calcification and craniosynostosis have been observed in our patient, further careful investigations are necessary.

The change in circulating TNSALP substrate levels in our patient is an important consideration with regard to therapeutic efficacy. In our patient, PLP levels declined during administration of AA therapy, but remained within the upper limit of the reference range, whereas the PPi level at baseline was within the reference range and did not show any apparent changes related to AA therapy. A Phase II open-label study of infants and young children with HPP showed that PLP and PPi decreased to reach the reference range following 6 months of treatment with standard-dose AA therapy (31). Additionally, in a study of adults and adolescents with pediatric-onset HPP, AA therapy at lower doses (2.1–3.5 mg/kg/week) led to normalization of circulating PLP and PPi levels, as well as significant improvement in functional assessments such as the 6-Minute Walk Test; however, the reduction in PPi did not reach statistical significance. These findings indicate that lower doses of AA might be suboptimal with respect to reduction of TNSALP substrate (18), in the same way as our patient. Unfortunately, the levels of PLP and PPi were reported after a delay (upon request to Alexion Pharmaceuticals Inc.); thus, we could not use these values as indicators of treatment-responsiveness. Furthermore, we cannot fully investigate changes in PLP and PPi levels due to current unavailability of commercial measurement assays in Japan. It is therefore important to identify good markers for assessment of responsiveness to AA therapy.

In conclusion, in our patient, early intervention with AA therapy presumably prevented the progression of HPP; furthermore, adjusted lower doses of AA were sufficient for maintenance of improved skeletal features. We cannot conclude that our modified regimen is an alternative (or improved) approach for patients with HPP who lack apparent severe clinical symptoms; however, our findings highlight the potential feasibility of a tailored enzyme replacement therapy approach based on clinical severity in patients with HPP.

## Patient Perspective

Patient’s parents have been totally agreed with publishing the report describing their daughter’s case. They have fully understood the reasons for applying the modified regimen of AA therapy to their daughter.

## Data Availability Statement

The original contributions presented in the study are included in the article, further inquiries can be directed to the corresponding author.

## Ethics Statement

The studies involving human participants were reviewed and approved by the Independent Ethics Committee of Hamamatsu University School of Medicine. Written informed consent was obtained from the minor(s)’ legal guardian/next of kin for the publication of any potentially identifiable images or data included in this article.

## Author Contributions

All coauthors read and met the *Frontiers in Endocrinology* criteria for authorship. YF wrote the manuscript. TK performed the direct sequence analysis. All authors listed have made a substantial, direct, and intellectual contribution to the work, and approved it for publication. All authors contributed to the article and approved the submitted version.

## Funding

This study was supported by a Grant for Research on Intractable Diseases from the Ministry of Health, Labour and Welfare (H27–025).

## Conflict of Interest

The authors declare that the research was conducted in the absence of any commercial or financial relationships that could be construed as a potential conflict of interest.
